# Analytical Model of Two-Directional Cracking Shear-Friction Membrane for Finite Element Analysis of Reinforced Concrete

**DOI:** 10.3390/ma14061460

**Published:** 2021-03-17

**Authors:** Jeffrey P. Mitchell, Bum-Yean Cho, Yoo-Jae Kim

**Affiliations:** 1 Department of Civil Engineering, Washington University in St. Louis, 1 Brookings Drive, Saint Louis, MO 63130, USA; jmleeedge@gmail.com; 2 Department of Fire Safety Research, Korea Institute of Civil Engineering and Building Technology, 64 Ma-doro, 182 Beon-gil, Mado-myeon, Hwaseong-si, Gyeonggi-do 18544, Korea; choby277@kict.re.kr; 3 Department of Engineering Technology, Materials Science, Engineering, and Commercialization Program, Texas State University, 601 University Drive, San Marcos, TX 78666, USA

**Keywords:** shear friction, membrane, crack-opening path, finite element, modified Newton–Raphson method

## Abstract

There are a multitude of existing material models for the finite element analysis of cracked reinforced concrete that provide reduced shear stiffness but do not limit shear strength. In addition, typical models are not based on the actual physical behavior of shear transfer across cracks by shear friction recognized in the ACI 318 Building Code. A shear-friction model was recently proposed that was able to capture the recognized cracked concrete behavior by limiting shear strength as a yielding function in the reinforcement across the crack. However, the proposed model was formulated only for the specific case of one-directional cracking parallel to the applied shear force. This study proposed and generalized an orthogonal-cracking shear-friction model for finite element use. This was necessary for handling the analysis of complex structures and nonproportional loading cases present in real design and testing situations. This generalized model was formulated as a total strain-based model using the approximation that crack strains are equal to total strains, using the proportional load vector, constant vertical load, and modified Newton–Raphson method to improve the model’s overall accuracy.

## 1. Introduction

Over the past 30 years, an element-based approach was developed to study reinforced-concrete structures based on the premise that the behavior of any whole structure can be predicted by integrating what is learned from experiments about its parts or elements [1]. In many typical reinforced-concrete structures, such as shear walls, applied loads are resisted primarily by in-plane stresses, often referred to as membrane stresses. Therefore, each membrane element can resist two in-plane normal stresses and one in-plane shearing stress.

It is important to choose accurate and robust modeling techniques for finite-element analysis that can capture the desired behavior. The ultimate goal of finite-element modeling is to provide computationally efficient and stable formulations that are easy to implement and provide accurate and reasonable results. Just as in selecting constitutive relationships, there are differing methods and viewpoints for a finite model that accomplishes these goals. Two of these issues include stiffness formulations (secant or tangent) and crack models (rotating or fixed). Some of the most robust models were developed by Vecchio and Hsu [1,2].

There are several suggested methods for modeling shear stress–strain relationships in cracked concrete [3,4]. The constant shear modulus method is more commonly known as the shear retention factor. It is obtained by multiplying the full, elastic shear modulus of uncracked concrete by a shear retention factor ranging from 0 to 1. Use of the shear retention factor is inconsistent with all primary mechanical relationships of accurate shear models for cracked concrete [5]. More importantly, there is no rational basis for selecting the value of *β*, and it does not limit the shear strength. Zhu and Hsu [6] presented a rational shear modulus that is provided on a smeared level as a function of principle stresses and strains. This method assumes that the principle stress and strain directions coincide to simplify the shear modulus. Vecchio and Collins [2] proposed that the modified compression-field theory (MCFT) provides a smeared secant shear modulus as a function of principle stresses and strains, as well. Following the MCFT, Vecchio [7,8] presented that the disturbed stress field model (DSFM) was developed to handle some issues not considered in the MCFT. The DSFM theory included rigid-body concrete shear slip by allowing deviations of the principal stress and strain directions. Walraven [9] presented a paper on reinforced concrete crack surface when subjected to seismic shear forces. It is based on the locking of aggregate particles on a crack surface as a function of crack width and slip, as well as aggregate size and geometry. Then the shear-friction force developed into the normal compressive force on the crack surface provided by tension in the reinforcement crossing the crack.

So et al. [5] proposed a shear-friction model for the finite-element analysis of reinforced-concrete membranes, which sought to improve finite-modeling techniques for shear transfer across cracked reinforced concrete. This proposed theory is a rational model based on the physical behaviors present in the ACI 318 Code provisions for shear friction, including limiting the shear strength due to steel yielding.

This paper begins with the development of the orthogonal shear friction theory, (OSFT) which is inserted into the new finite-element framework. The model will be developed in five phases.

## 2. Research Significance

The current proposed shear-friction theory is a rational model based on the physical behaviors present in the ACI building code. The shear-friction model is capable of handling only a precracked element, with only one crack direction. In addition, the crack direction is oriented in the direction of the applied shear. This paper will develop and formulate the two-directional orthogonal-cracking shear-friction model for the finite-element framework, including five phases of the model’s development.

## 3. Brief Description of the Shear-Friction Model

**Figure 1:**
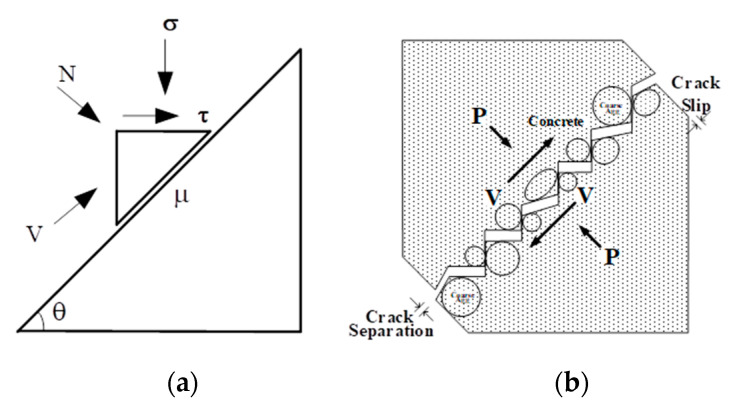
(**a**) Concept of crack slip mechanism and (**b**) Crack opening path [15].

The mechanical relationships of shear-friction behavior present in ACI 318 [10] were the basis for formulating the initial concept. Previous testing and data was reviewed to develop a basic mechanical model of the behavior of slip across a crack [11]. The friction developed on the surface of the crack can then be defined by the normal and shear forces acting on the crack surface, as shown in Figure 1, in which the friction coefficient (*μ*) is equal to *τ/σ*. However, further evaluation of previous testing showed that the coefficient of friction relative to the crack surface varied considerably with the amount of slip, an approximate value of between 0.6 and 0.7, which showed that it was more suitable to define the coefficient of friction in terms of the crack-opening path [11,12,13,14].

In strain space, the smeared crack strains (*ε_cr_* and *γ_cr_*) are the crack separations and slips divided by the average crack spacing. This definition of smeared crack strains only works if the crack-opening path is simplified to a linear relationship, otherwise the crack shear and normal stresses would not be uniquely determined by the crack strains. Crack slip can be compared with pushing a block up a slope with a rough surface, in which the coefficient of friction relative to the crack-opening path can be defined. The friction coefficient relative to the surface of the slope is defined by Equation (1) [15]:

(1) $$\mu^{\prime }=\frac{N}{V}$$

This definition of the friction coefficient results in values that vary little in relation to slip. It also allows for a method in computing a friction coefficient for both directions of slip because pushing a block up a slope is more difficult than pushing it down a slope. The friction coefficients *μ^up^* and *μ^down^* are defined by Equations (2) and (3) for either the upslope or downslope direction as functions of the single-crack-path friction coefficient *μ’* and the angle of the crack opening path *θ* (both should remain constant) [5]. The detailed process of the one-crack formulation is referred to in [16].

(2) $$\mu^{up}=\frac{\left[\sin\theta +\mu^{\prime }\cos\theta \right]}{\left[\cos\theta -\mu^{\prime }\sin\theta \right]}$$

(3) $$\mu^{down}=\frac{\left[\mu^{\prime }\cos\theta -\sin\theta \right]}{\left[\cos\theta +\mu^{\prime }\sin\theta \right]}$$

## 4. Proposed Orthogonal-Cracking Model Based on Shear Friction

A linear crack-opening path defined by the total shear strain and the total normal strain relative to the crack direction will be assumed to enforce the slip–separation relationship, such that *γ = a_cop_ε*. The crack-path model is a constitutive model for concrete subjected to uniaxial compression that predicts axial and transverse strains as functions of the applied axial stress. It assumes the presence of closely spaced slip surfaces that occur at all possible planar orientations that form an angle approximately equal to 0.45 radians with respect to the axis of loading. Strain is the sum of linear elastic strain in the material between slip surfaces and nonlinear strain due to slip and separation of the surfaces. For convenience, the slip surfaces can be thought of as closely spaced cracks. Shear and normal strain equal the slip and separation divided by the average crack spacing, respectively. Slip causes the crack to separate due to crack-surface roughness. The relationship between slip and separation is referred to as the “crack-opening path” for convenience. The slope of the crack-opening path can flatten due to stress normal to the crack surface.

The parameters of the crack-path model are the crack-opening-path model, the relationship between tension stress normal to the crack surface and crack separation (see Figure 1), and the crack-friction coefficient. The parameters are different than the traditional friction coefficient in that they are defined relative to the slope of the crack-opening path, rather than to the crack surface. In this way, a constant friction coefficient is obtained, rather than one that varies with the slope of the crack-opening path. In this approach, the effects of friction and dilatancy are separated and the friction coefficient (*µ′*) becomes approximately constant. The parameter *a_cop_* is a crack-opening-path slope that can be estimated from shear-friction test and a parametric study. See [15,16] for more details. From the crack-opening path, the effective strain *^e^ε* can be defined. For the initial formulation of the model, it is assumed that the total strains are almost all crack strains. Total shear slip will be tracked as it will be equal to the crack slip. Material properties and cracks are also smeared across the element. Once two cracks have formed, it will be difficult to determine how incremental shear strain is distributed between the two crack directions. Another important assumption will be that only one crack can slip or be “active” at a time.

### 4.1. Stress/Strain Formulations

Bilinear, rectangular, simple four-noded elements were chosen that had 8 degrees of freedom (DOFs) and could translate horizontally and vertically.

**Figure 2:**
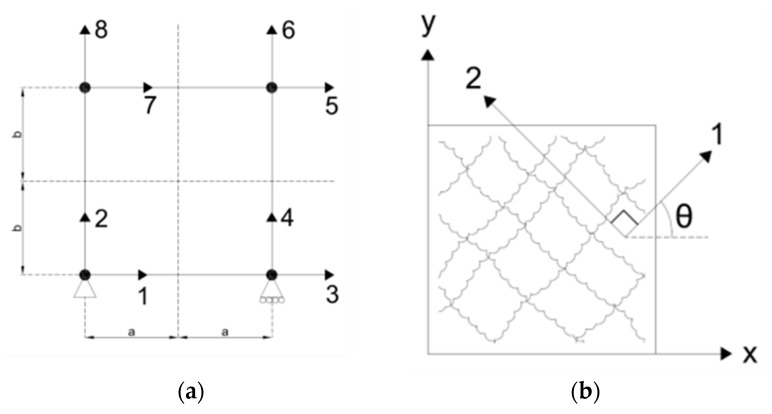
(**a**) Bilinear rectangular element, dimensions, DOFs and boundary conditions. (**b**) Element coordinate systems.

Figure 2a shows the element, its dimensions, the DOFs, and the boundary conditions. The element is square with dimensions “2a” and “2b”, and a thickness “t” where in this case, a = b. Notations a and b make up half of the length of an element. The bottom-left node is pinned (restrained both vertically and laterally), and the bottom right node serves as a roller, free to translate laterally only. All of the odd-numbered DOFs represent lateral translation of the nodes, while the even numbered DOF’s represent vertical translation. Figure 2b shows the element’s coordinate definitions. The global coordinate system is “x–y” and the local crack-direction coordinate system is “1–2”, which is defined by angle *θ*. The 1-direction is defined to be orthogonal to the first crack.

**Figure 3:**
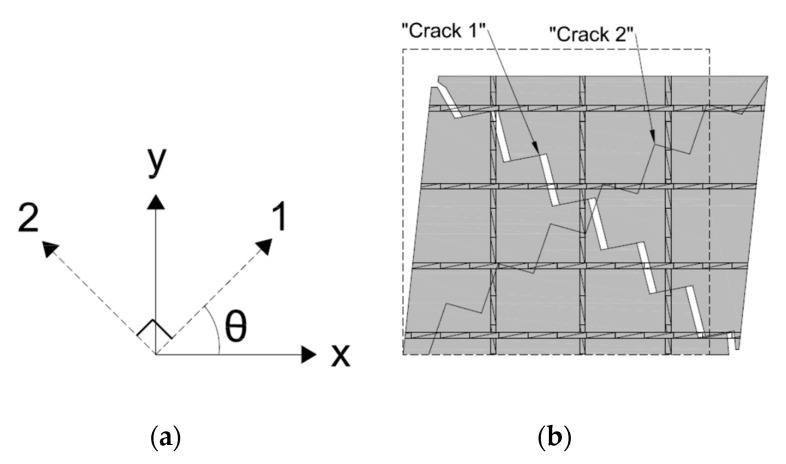
(**a**) Crack definitions and (**b**) Schematic of the slip–separation relationship of the active crack.

The first crack will be labeled “Crack 1” and set as the local crack coordinates “1–2” as shown in Figure 3. Figure 3 also shows the element in a deformed state with Crack 1 open. Both cracks are in compression, but only Crack 1 has slipped, inducing separation across the surface, while Crack 2 remains in compression and has zero slip. Although Crack 2 has neither slipped nor separated, in theory it could still be the active crack in a state of “no slip”. For the initial formulation of the OSFT stress–strain relationships, only Crack 1 will be considered active.

#### 4.1.1. Effective Strain

For orthogonal cracking, the epsilon effective is defined for each crack. Equations (4) and (5) define effective strain by subtracting the separation strain due to crack slip (crack opening path in the 1–2 direction) from the total strain normal to the crack:

(4) ε1e=ε1−|γ12|acop

(5) ε2e=ε2−|γ12|acop

#### 4.1.2. Normal Concrete Stress

Effective strain is used to define the concrete stresses normal to the crack surfaces *σ*_1_ and *σ*_2_. Positive values of effective strain represent tension across the crack surface, while negative values represent compression. A tension-stiffening curve is used to model the reduced stiffness in the tensile strain region, and the full elastic modulus of concrete is used for simplicity in the compressive strain regions. Equations (6) and (7) [5] for normal stress in the concrete are listed below, formulated for Crack 1. Equations for Crack 2 can be obtained by replacing “1” with “2” for the stress and strain notations.

(a) *^e^ε*_1_*≤ ε_cr_* (the cracking strain of concrete):
  (6) σ1=Ecε1e=Ec(ε1−|γ12|acop)
(b) *^e^ε_1_ > ε_cr_*:
  (7) σ1=fcr1+200ε1e

#### 4.1.3. Maximum and Minimum Shear Stresses

The shear-stress equations are combined into two major categories: when normal stress to the crack is tensile, and when normal stress to the crack is compressive. Within the compressive normal stress category, shear stress is broken down again into two categories: positive and negative shearing strain relative to the crack. At this point, limits on the stresses are introduced to distinguish the physical behavior of the crack. At any given point, the crack surface could be slipping up the slope, down the slope, or not slipping at all. All of the equations presented here assume that Crack 1 is the active crack.

- Tension across the Crack

If the normal stress *σ*_1_ is tensile, shear stress is calculated as follows, assuming that this is the shearing stress resisted by dowel action of the reinforcement crossing the crack:

(8) $$\tau_{12}=\frac{\beta^{\prime }G\gamma_{12}}{\left(1+\beta^{\prime }\right)}$$

The premise is that there is some minimal stiffness when the cracks are not in contact.

- Compression across the Crack

If normal stress *σ*_1_ is compressive and the crack surfaces are in contact, shear stress is determined by the equations shown in Table 1.

For positive shearing strain (*γ*_12_ > 0) and negative shearing strain (*γ*_12_ < 0), the three cases considered are shown in Table 1: if the crack surface is slipping down a slope, “if the crack surface is not slipping and if the crack surface is slipping up a slope.

For any given shear strain *γ*_12_, shear stress *τ*_12_ is determined from the “not slipping” equation, but is limited by the minimum and maximum shear stresses, *τ*^a^_12_ and *τ*^c^_12_, as shown below:

$$\tau_{12}=\tau_{12}^{b}$$

$\mathrm{where},\;\tau_{12}^{a}\leq \tau_{12}\leq \tau_{12}^{c}$.

These shear-stress equations can be formulated for Crack 2 by replacing the normal stress *σ*_1_ with the normal stress relative to Crack 2, *σ*_2_.

#### 4.1.4. Secant Stiffness Matrix Formulation

The base secant stiffness matrix used to define the concrete stress–strain relationship is a 3 × 3 diagonal matrix, as shown below. This matrix will be altered, however, to include the complete OSFT, including effective strain, tension stiffening, the crack opening path and maximum and minimum shear stresses.

(9) $$\left\{\begin{array}{c} \sigma_{1}\\ \sigma_{2}\\ \tau_{12} \end{array}\right\}=\left[\begin{array}{ccc} \frac{\sigma_{1}^{old}}{\varepsilon_{1}^{old}} & 0 & 0\\ 0 & \frac{\sigma_{2}^{old}}{\varepsilon_{2}^{old}} & 0\\ 0 & 0 & \frac{\tau_{12}^{old}}{\gamma_{12}^{old}} \end{array}\right]\left\{\begin{array}{c} \varepsilon_{1}\\ \varepsilon_{2}\\ \gamma_{12} \end{array}\right\}$$

**Case 1: Crack Direction 1 Active**

Once Crack 1 has formed, cases for stiffness must be considered:

(a) If the normal stress is tensile, the stress–strain relationship is as follows for positive and negative shearing strain, *γ*_12_:
  (10) $$\left\{\begin{array}{c} \sigma_{1}\\ \sigma_{2}\\ \tau_{12} \end{array}\right\}=\left[\begin{array}{ccc} E_{1} & 0 & \frac{\pm E_{1}}{a}\\ 0 & E_{2} & 0\\ 0 & 0 & \frac{\beta^{\prime }G}{1+\beta^{\prime }} \end{array}\right]\left\{\begin{array}{c} \varepsilon_{1}\\ \varepsilon_{2}\\ \gamma_{12} \end{array}\right\}$$
  where *E*_1_ is the secant stiffness of concrete defined as the normal stress (Equation (7)) divided by the current effective strain value, ^e^ε_1_. *E*_2_ can be obtained in the same manner as *E*_1_ by replacing “1” with “2” in the stress and strain notations.
(b) If normal stress is compressive, the secant stiffness matrix is defined based on normal compressive stress (Equation (6)) and the various cases of shear stress. For positive total shear strain (*γ*_12_ > 0) and negative total shearing strain (*γ*_12_ < 0), the equations and secant stiffness matrices can be found in Table 2 and Table 3.

**Case 2: Crack Direction 2 Active**

Once Crack 2 has formed, the following cases for stiffness must be considered if Crack 2 is the active crack:

(a) If normal stress is tensile, the stress–strain relationship is as follows for positive and negative shearing strain, *γ*_12_:
  (11) $$\left\{\begin{array}{c} \sigma_{1}\\ \sigma_{2}\\ \tau_{12} \end{array}\right\}=\left[\begin{array}{ccc} E_{1} & 0 & 0\\ 0 & E_{2} & \frac{\pm E_{2}}{a}\\ 0 & 0 & \frac{\beta^{\prime }G}{1+\beta^{\prime }} \end{array}\right]\left\{\begin{array}{c} \varepsilon_{1}\\ \varepsilon_{2}\\ \gamma_{12} \end{array}\right\}$$
(b) If normal stress is compressive, the secant stiffness matrix is defined based on the normal compressive stress and the various cases of shear stress. For positive total shear strain (*γ*_12_ > 0) and negative total shearing strain (*γ*_12_ < 0), secant stiffness matrices can be found in Table 4.

**Active Crack Criteria**

The OSFT includes 16 different stiffness formulations to model the concrete behavior at any given state of stress and strain for two crack directions. These 16 formulations become pertinent once orthogonal cracking has occurred. In order to implement the OSFT into a finite-element program, rational criteria for an active crack and the current state of the crack surface needs to be established.

At any given state of stress and strain, the crack surface may be in tension or compression. This is defined by the crack-opening path, specifically effective strain. Positive values of effective strain represent tension across the crack surface, meaning that the rough surfaces of the crack are not in contact. Negative values of effective strain represent compression across the crack, meaning that the rough surfaces of the crack are in contact and shear friction can be developed.

If both cracks are in tension, then crack slip (or *γ*_12_) is split between the cracks. However, this case is unlikely for most realistic loading situations. If Crack 1 is in tension and Crack 2 is in compression, then the active crack is defined as Crack 1. This is because slip would tend to be focused on the open crack, as it can only be resisted by tension in the reinforcement crossing the crack. If Crack 2 is in tension and Crack 1 is in compression, however, the active crack will be Crack 2. The last possible case is both cracks being in compression. To determine the active crack for this situation requires the maximum and minimum shear-stress limits. The upper and lower limits on shear stress (*τ^a^* and *τ^c^*) represent the stress necessary to overcome the force of friction across the crack and initiate slip.

**Figure 4:**
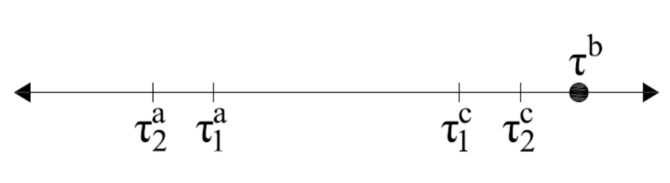
Example shear-stress limits on a one-dimensional plot.

Shear-stress limits for both cracks are necessary to determine the active crack (*τ^a^*_1_*, τ^a^*_2_*, τ^c^*_1_*,* and *τ^c^*_2_). The active crack will be defined as the crack that has reached the limits first, because slip would be initiated in this crack first, thus controlling the behavior. The shear-stress limits on a one-dimensional plot are shown in Figure 4. A summary of the active crack criteria is presented in Table 5.

### 4.2. Uniform State of Stress and Strain

A uniform state of strain is defined as strain that remains consistent across the dimensions of the element. For a rectangular element, like the membrane being analyzed in this study, straight lines before strain remain straight after strain, and parallel lines before strain remain parallel after strain. Thus, a rectangular element subjected to uniform strain could deform into a parallelogram. A uniform state of stress follows suit, as stresses will be consistent across the dimensions of the element.

Without a uniform state of stress and strain, analyzing and interpreting the results would become cumbersome, and critical material behavior may be lost. Obtaining a uniform state of stress and strain consequently becomes a major constraint on the design of the finite-element program.

#### Shear Stress across the Crack Surface

**Figure 5:**
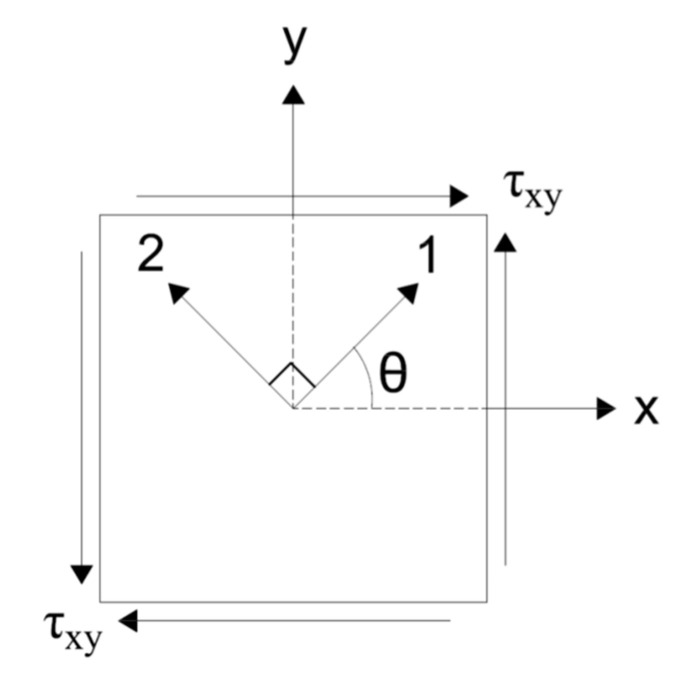
Element subjected to pure shear.

To generalize the shear-friction model to include orthogonal cracking at any angle, the concrete cannot be precracked, but should be allowed to crack in the direction of the principal stresses. Considering an element subjected to pure shear as shown in Figure 5, the orientation of the principal stresses 1 and 2 will be at an angle *θ* of 45° with respect to the x–y coordinate system. In this case, the 1-direction is the principal tensile stress, thereby creating a crack parallel to the 2-direction. The shear stress transformed at an angle of 45° to the x–y coordinate system will be equal to zero. With no shear across the surface of the crack, transfer by shear friction becomes irrelevant. The only way to obtain shear across the crack is to apply normal stress to the element.

## 5. Developmental Study of the Finite-Element Model

There were five phases in the development process of the finite-element model for the OSFT.

### 5.1. Phase 1: Nonlinear Cyclical Model Framework

**Figure 6:**
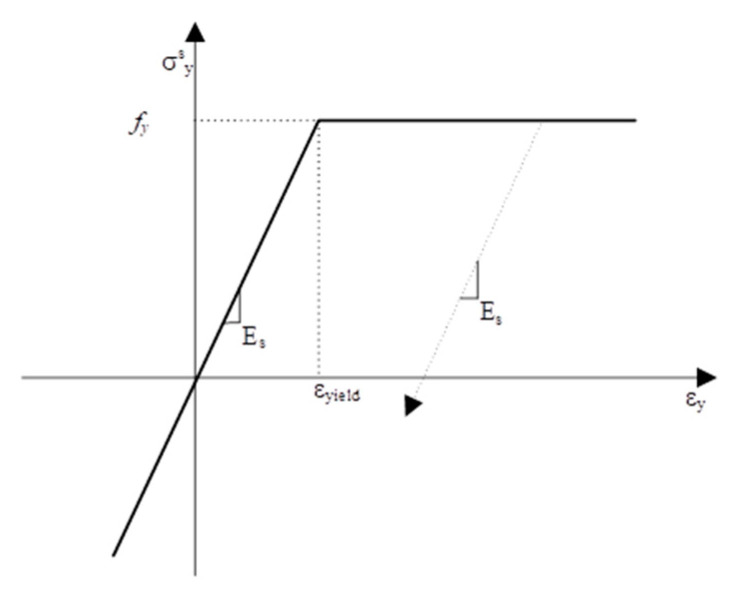
Nonlinear steel stress–strain relationship.

The first step toward a working orthogonal-cracking shear-friction model was to produce a nonlinear finite-element framework, which was an analysis of a single bilinear rectangular element simply supported on the bottom two nodes. This initial framework included nonlinear steel and concrete material properties previously established in stress–strain formulations, including the tension-stiffening curve [16]. The nonlinear steel model presented in Figure 6 was kept consistent in all phases of the model development.

The smeared steel stress is simply the strain multiplied by the steel modulus of elasticity multiplied by the reinforcement ratio until the steel yields. Once the steel yields, the smeared stress is equal to the yield strength of the steel multiplied by the reinforcement ratio. For unloading the steel from the maximum excursion strain, the slope of the steel modulus of elasticity is used.

### 5.2. Phase 2: Initial Shear-Friction Implementation

The OSFT was inserted into the initial finite-element framework. Displacements were incremented monotonically until the strain was sufficient to crack the concrete. As previously stated, the displacements were enforced to create a uniform state of stress and strain. Once the crack angle was established, the OSFT was activated to define the concrete stress–strain relationships and stiffness. These relationships were formulated in the crack coordinate system (1–2 direction), then rotated to the global coordinate system (x–y direction) for analysis.

### 5.3. Phase 3: Proportional Load Vector (PLV)

To obtain shear across the crack surface and apply a compressive vertical load on the element, a proportional load vector was used to obtain the desired displacements. The proportional load vector ensured a uniform state of stress and strain, while still allowing a displacement controlled analysis. Formulations of the PLV, a new convergence method, results and difficulties are presented in the subsections of this section.

For a displacement-controlled analysis, the global stiffness matrix was previously partitioned into the free, restrained and prescribed degrees of freedom as shown below.

(12) $$\left\{\begin{array}{c} A_{F}\\ A_{P}\\ A_{R} \end{array}\right\}=\left[\begin{array}{ccc} S_{FF} & S_{FP} & S_{FR}\\ S_{PF} & S_{PP} & S_{PR}\\ S_{RF} & S_{PR} & S_{RR} \end{array}\right]\left\{\begin{array}{c} D_{F}\\ D_{P}\\ D_{R} \end{array}\right\}$$

**Figure 7:**
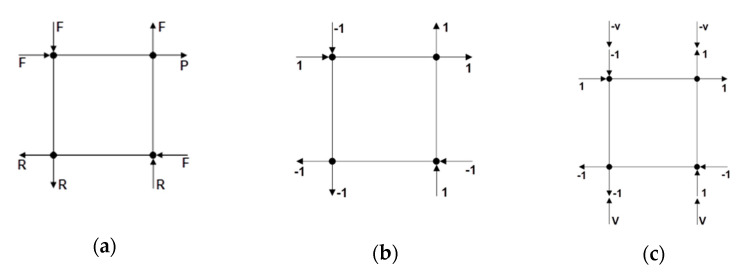
(**a**) Partitioned DOFs for the PLV. (**b**) Shape vector applied to the element. (**c**) Shape vector and vertical load, v, applied to element.

The PLV formulation similarly requires partitioning the global stiffness matrix into free, prescribed and restrained DOFs. As shown in Figure 7a, however, the partitioned DOFs were slightly different. The PLV states that there is a shape vector (Ã) that defines the shape of the loads on the DOF such that:

(13) A=Ãa

To ensure a state of uniform stress and strain, the shape vector is defined as follows:

$$Ã=\left\{\begin{array}{c} -1\\ \begin{array}{c} -1\\ -1\\ \begin{array}{c} \;\;\;1\\ \;\;\;1\\ \begin{array}{c} \;\;\;1\\ \;\;\;1\\ -1 \end{array} \end{array} \end{array} \end{array}\right\}where\;the\;DOF^{}s\;are\;:\;\begin{array}{c} 1\\ 2\\ 3\\ 4\\ 5\\ 6\\ 7\\ 8 \end{array}$$

Figure 7b shows the shape vector applied to the element’s DOFs. By applying Equation (13) to the global stiffness matrix, and performing the matrix multiplication, the following equations can be written:

(14) $$a{Ã}_{F}=S_{FF}D_{F}+S_{FP}D_{P}+S_{FR}D_{R}$$

(15) $$a{Ã}_{P}=S_{PF}D_{F}+S_{PP}D_{P}+S_{PR}D_{R}$$

(16) $$a{Ã}_{R}=S_{RF}D_{F}+S_{RP}D_{P}+S_{RR}D_{R}$$

Applying the boundary conditions (*D_R_* = 0), plugging Equation (14) into Equation (15) and solving for *a* yields:

$$a=\frac{S_{PP}D_{P}-S_{PF}S_{FF}{}^{-1}S_{FP}D_{P}}{{Ã}_{P}-S_{PF}S_{FF}{}^{-1}{Ã}_{F}}$$

The solution for *a* is driven by the prescribed displacement, i.e., the PLV still allows for a displacement-controlled analysis. To accomplish this, a vertical load (v) can be applied to the shape vector as shown in Figure 7c.

Now the shape vector can be defined as follows:

$$Ã=\left\{\begin{array}{c} -1\\ \begin{array}{c} -1+v\\ -1\\ \begin{array}{c} \;\;\;1+v\\ \;\;\;1\\ \begin{array}{c} \;\;\;1-v\\ \;\;\;1\\ -1-v \end{array} \end{array} \end{array} \end{array}\right\}where\;the\;DOF^{}s\;are\;:\;\begin{array}{c} 1\\ 2\\ 3\\ 4\\ 5\\ 6\\ 7\\ 8 \end{array}$$

The solution for *a* remains the same, and the vertical load (v) can be defined to ensure compression across the crack surface. The vertical compression applied to the element is not constant in this formulation, but will change depending on the solution for *a*.

**Figure 8:**
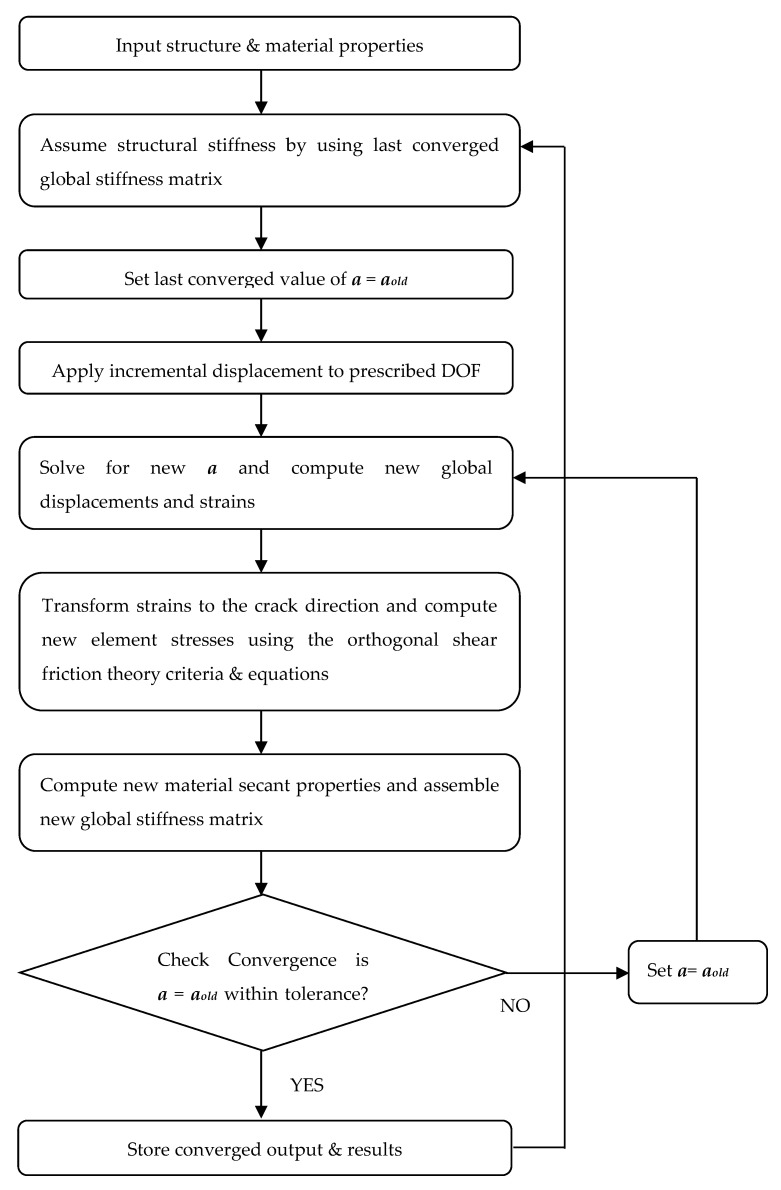
Flowchart of the PLV algorithm.

With the new formulation of the PLV, it is convenient to converge on the constant (*a*) while still updating the material secant values within an iteration. Figure 8 shows a flowchart of the PLV algorithm to illustrate the convergence method.

### 5.4. Phase 4: Constant Vertical Load (CVL)

The PLV formulation applied a net vertical compressive load to the element. However, the vertical compressive load is proportional with *a*, increasing and decreasing with *a*. In order to resolve this, the vertical compressive load needs to be independent of *a*. This is also more reasonable, as realistic structures generally have a constant vertical load for design, not a variable one. To accomplish this, Equation (13) was modified as follows:

(17) $$A=Ãa+\;\tilde{B}$$

$$where,\;Ã=\left\{\begin{array}{c} -1\\ -1\\ -1\\ \;1\\ \;1\\ \;1\\ \;1\\ -1 \end{array}\right\}and\;\;\tilde{B}=\left\{\begin{array}{c} \;\;\;0\\ \;\;\;0\\ \;\;\;0\\ \;\;\;0\\ \;\;\;0\\ -v\\ \;\;\;0\\ -v \end{array}\right\}\;and\;the\;DOF^{}s\;are:\;\begin{array}{c} 1\\ 2\\ 3\\ 4\\ 5\\ 6\\ 7\\ 8 \end{array}$$

In addition, Equation (14) through Equation (16) need to be modified to account for the changes to Equation (13). The DOFs and the two load vectors (Ã and $\tilde{B}$) will be partitioned again into free, prescribed and restrained portions as follows:

(18) $$a{Ã}_{F}+{\tilde{B}}_{F}=S_{FF}D_{F}+S_{FP}D_{P}+S_{FR}D_{R}$$

(19) $$a{Ã}_{P}+{\tilde{B}}_{P}=S_{PF}D_{F}+S_{PP}D_{P}+S_{PR}D_{R}$$

(20) $$a{Ã}_{R}+{\tilde{B}}_{R}=S_{RF}D_{F}+S_{RP}D_{P}+S_{RR}D_{R}$$

By applying the boundary conditions, Equation (20) can be solved for *D_F_* and plugged into Equation (19). Now *a* can be solved, such that:

$$a=\frac{S_{PF}S_{FF}{}^{-1}\left({\;\tilde{B}}_{F}-S_{FP}D_{P}\right)+S_{PP}D_{P}}{{Ã}_{P}-S_{PF}S_{FF}{}^{-1}{Ã}_{F}}$$

**Figure 9:**
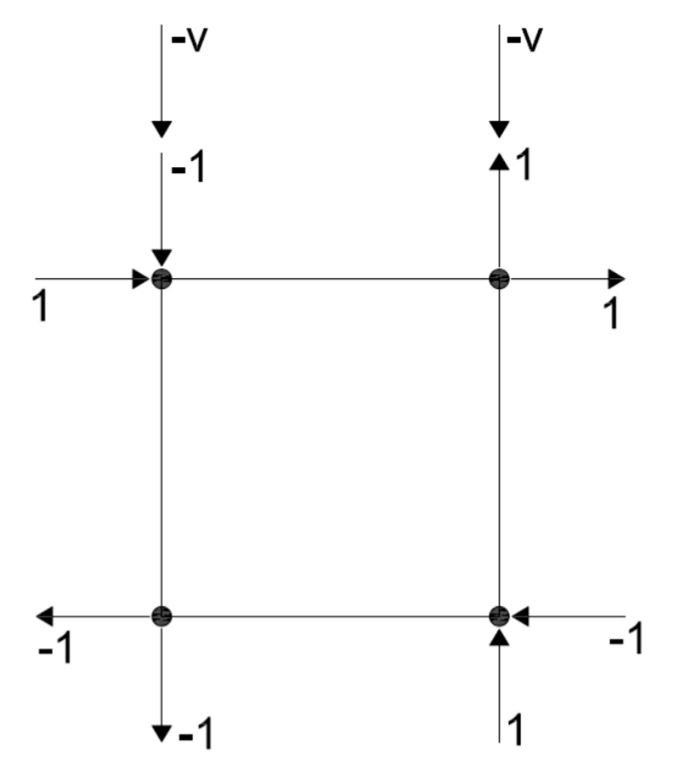
Diagram of loading for the CVL formulation.

It should be noted again that the solution for *a* is driven by the prescribed displacement, i.e., the addition of the constant load vector still allows for a displacement-controlled analysis. The convergence scheme and program algorithm were the same as the PLV. Figure 9 diagrams the loading imposed on the element from the CVL formulation.

### 5.5. Phase 5: Modified Newton–Raphson Method (MNRM)

In an attempt to obtain convergence over cyclical loading of the current model, a new convergence algorithm was developed. The MNRM works by releasing and re-enforcing the prescribed DOF within an individual convergence iteration until the forces are consistent with the state of stress. The new convergence algorithm was applied to the CVL model, preserving the loading, shear-friction parameters, general analysis procedures and other inputs.

**Figure 10:**
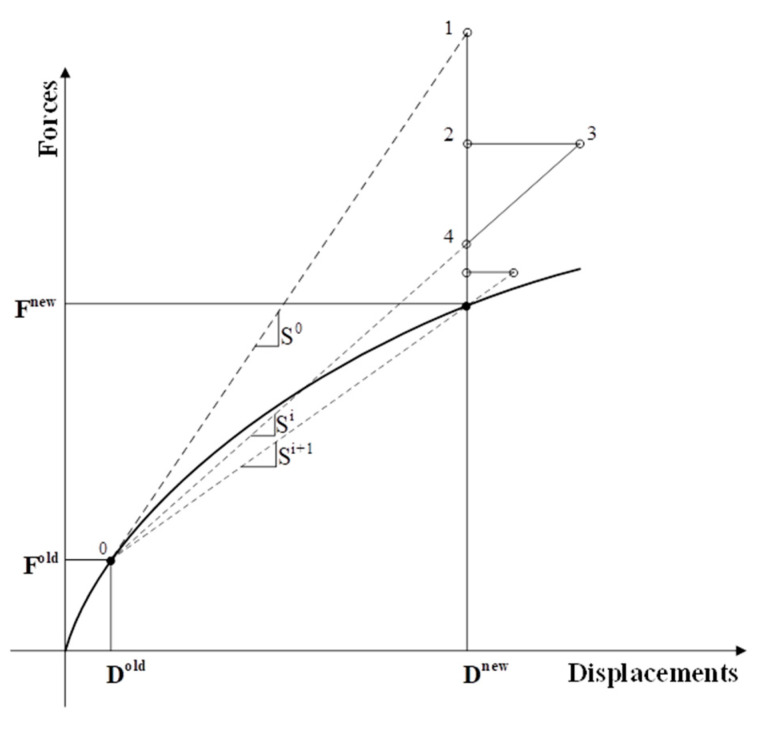
Procedure for the modified Newton–Raphson convergence method.

The procedure for the MNRM can be seen in Figure 10. The algorithm is based on ensuring consistency between the forces and the state of stress. The procedure starts at the last set of converged displacements and forces D^old^ and F^old^, respectively. This point “0” lies on a representative curve of the force–displacement relationship, which includes all of the model’s properties contained within the global stiffness matrix (S).

**Figure 11:**
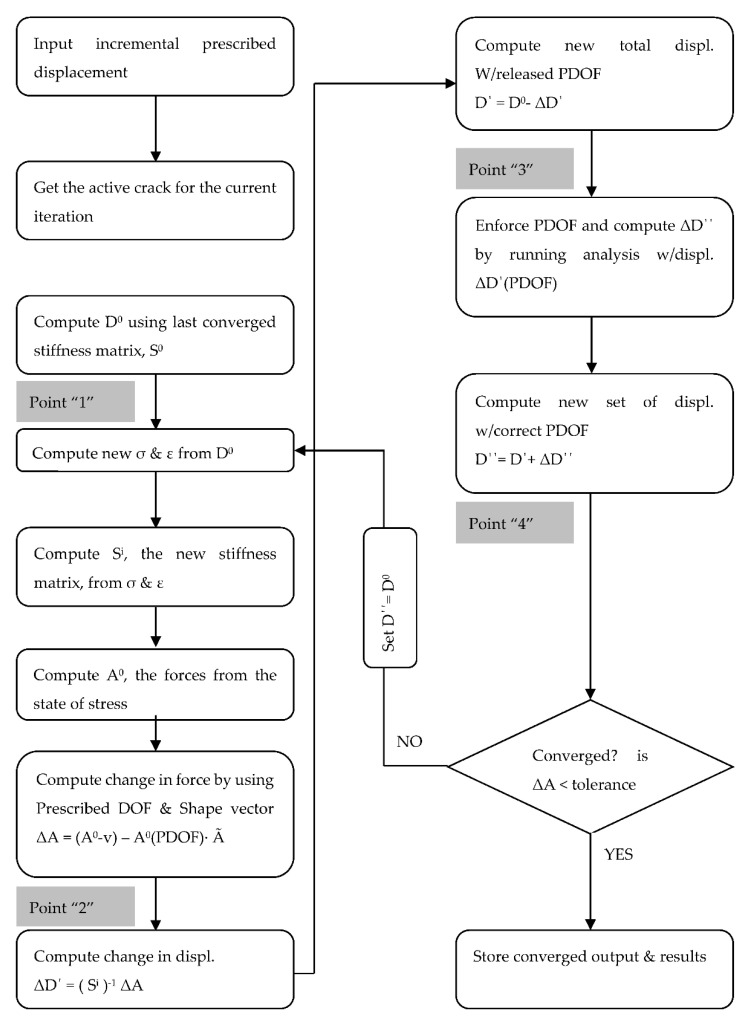
Flowchart of the modified Newton–Raphson algorithm.

From point “0”, a new incremental displacement is enforced, and the strains and stresses from this last converged point will define the state of the crack. The state of the crack defines which crack is active, forcing the program to only allow that crack to be active for the current iteration. As the state of active crack could, in theory, change within an iteration, the increment of enforced displacement will be made small enough to obtain reasonable results. Figure 11 shows an iterative approach for MNRM used.

## 6. Results and Discussions

A single four-node rectangular element was analyzed under the loading imposed by the proportional load vector. The concrete membrane was 254 mm tall by 254 mm wide and 51 mm thick. The reinforcement ratio was 2% in the horizontal direction and 0.5% in the vertical direction. The concrete was precracked at an angle (*θ*) of 45° setting the orthogonal cracking direction (1–2 direction). The vertical load (v) was initially set equal to 8.9 KN. The values of the base model material properties and parameters are shown in Table 6.

**Figure 12:**
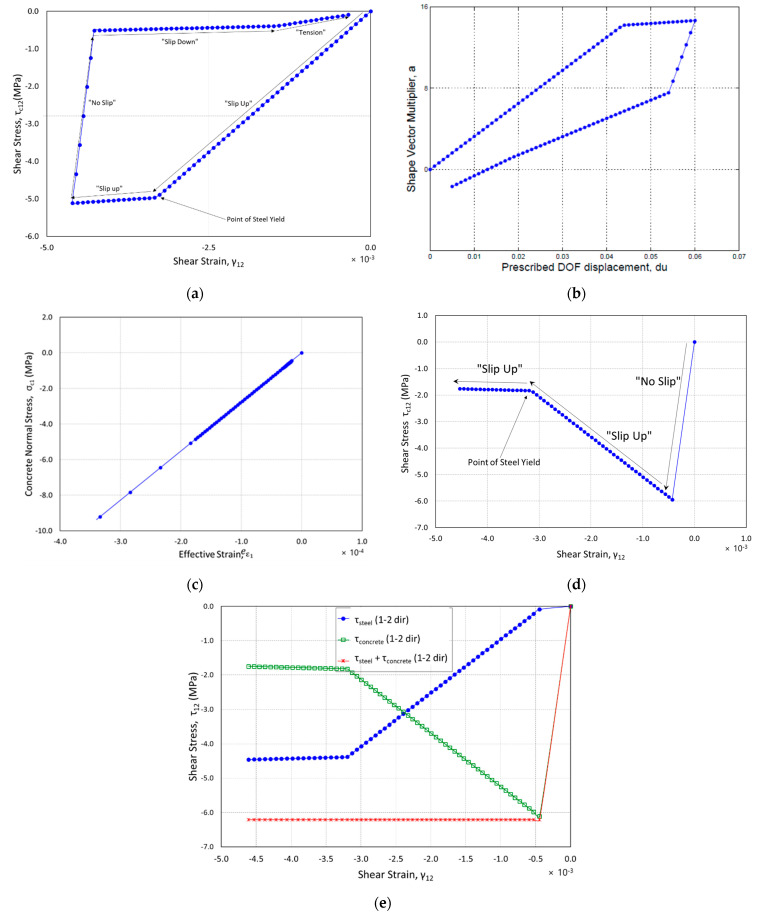
(**a**) Concrete shear stress (Crack 1) vs. shear strain (PLV); (**b**) shape vector multiplier vs. prescribed displacement (PLV); (**c**) concrete normal stress (Crack 1) vs. effective strain (CVL); (**d**) concrete shear stress (Crack 1) vs. shear strain (MNRM); (**e**) interaction between concrete and steel shearing stresses (MNRM).

Results of the analysis are presented in Figure 12a,b. The analysis was successful in loading and unloading in one direction, and the element was incrementally forced to displace to 1.52 mm in the x-direction at the prescribed DOF. Throughout the analysis only Crack 1 was the active crack.

Figure 12a shows that as displacements were enforced and shear slip occurred across the crack surface, the crack began to “slip up the slope” in the negative direction. This represents the stiffness attributed to the *β′G* portion of the “slip up” equation, and the shear friction force associated with “slip up”. Shear-friction resistance was due to the fact that as the crack surface slipped, it separated, causing tensile stresses in the steel. The tension in the reinforcement placed compression on the crack surface, causing shear-friction stress to develop. This shear-friction stress was directly proportional to the increasing compressive stress caused by the reinforcement. Once the steel was strained to its yield point, compressive stresses could no longer be increased across the crack surface, while the only increase in shear stress was due to the *β′G* term. This can be seen as the slope of the curve begins to flatten out.

Figure 12b shows the relationship between converged values of *a* in Equation (12), the multiplier of the shape vector, and the displacement (*du*). Only positive values for *a* yielded the correct forces in the proportional load vector, as the element was subjected to vertical tension once *a* exceeded the value of the applied load (v). This yielded an undesirable state of stress as the crack surfaces were always in tension, and shear friction could no longer be developed. This “flipped” state of *a* began to occur as the displacement approached zero.

A single four-node rectangular element was analyzed under the loading imposed by the CVL. All of the material properties were kept the same as in the PLV analysis. The reinforcement ratio was 2% in the horizontal direction and 0.2% in the vertical direction. The constant vertical load (v) was set equal to 80 KN. Results of the analysis are presented in Figure 12c–e.

Figure 12c shows the relationship between shear stress and shear strain in the crack orientation for loading in one direction. As the analysis began, shear stress in the concrete began to lessen as the crack surface “slipped up the slope”, which was due to the steel and concrete interaction for resisting shear in the direction of the crack. Figure 12d illustrates that the sum of the concrete and shear stresses remained constant. Initially, the concrete took almost all of the shear, as the “no slip” case provided the same shear stiffness of uncracked concrete. Once the crack surface began to slip, the concrete shear stiffness was reduced, and the steel began to resist more of the shear. Once the steel yielded, the curves flattened out, thereby limiting the shear strength.

Figure 12e shows how the normal stress was plotted against effective strain, and a realistic curve was obtained showing that the concrete model was enforced during the analysis. Based on the results and the new formulation, the reason for divergence might be due to the current convergence method, in that converging on *a* may not have been robust enough to handle the various nonlinear material properties.

The MNRM was successful in converging for loading in one direction. The analysis was run with the same inputs as the CVL formulation, and the results of the analysis were consistent with the results obtained from the CVL.

## 7. Conclusions

A new finite-element framework was required for the implementation of two-directional cracking. Because of this, a new framework was successfully developed, and results of the analysis showed that the shear-friction model was able to enforce the crack-opening path and limit the strength in shear by yielding in the reinforcement.

1. A deflection controlled analysis was successfully obtained in all phases of model development, which was necessary to produce useful and comparable results for arbitrary loading, such as cyclical loading.
2. A uniform state of stress and strain was successfully obtained in all phases of model development. It was first obtained by applying equal incremental displacements to the top two nodes. The top two nodes’ vertical displacements were then linked together. In later phases of the model development, a uniform state of stress and strain was obtained by a proportional load vector, ensuring this state while still allowing a displacement-controlled analysis.
3. Shear stress was successfully obtained across the surface of the crack. This was a major consideration, as the crack directions were defined by the orientation of the principal tensile stress. By definition, there is no shear in the direction of the principal stresses. Without shear being transferred across the surface of the crack, the consideration of shear friction for shear transfer becomes irrelevant. In order to obtain shear across the crack surface, a vertical compressive load was required. This was accomplished by means of a proportional load vector and later a constant vertical load combined with a proportional load vector.

The OSFT was developed and implemented in the finite-element framework. Due to convergence issues, however, only one crack was able to be analyzed, as the second crack was never stressed such that shear friction would be a consideration of stiffness. In an attempt to obtain convergence, the OSFT needs to be expanded to the crack strain separated theory, which would increase the model’s accuracy.

## Figures and Tables

**Table 1:** Equations for compression across the crack.

Shearing Strain	Crack Surface Slipping Down a Slope	Crack Surface Is Not Slipping	Crack Slipping Up a Slope
positive total shearing strain (*γ*_12_ > 0)	$\tau_{12}^{a}=\frac{\beta^{\prime }G\gamma_{12}+\mu^{down}\sigma_{1}}{\left(1+\beta^{\prime }\right)}$	$\tau_{12}^{b}=\tau_{12,prev}+G\left(\gamma_{12}-\gamma_{12,prev}\right)$	$\tau_{12}^{c}=\frac{\beta^{\prime }G\gamma_{12}-\mu^{up}\sigma_{1}}{\left(1+\beta^{\prime }\right)}$
negative total shearing strain (*γ*_12_ < 0)	$\tau_{12}^{c}=\frac{\beta^{\prime }G\gamma_{12}-\mu^{down}\sigma_{1}}{\left(1+\beta^{\prime }\right)}$	$\tau_{12}^{b}=\tau_{12,prev}+G\left(\gamma_{12}-\gamma_{12,prev}\right)$	$\tau_{12}^{a}=\frac{\beta^{\prime }G\gamma_{12}+\mu^{up}\sigma_{1}}{\left(1+\beta^{\prime }\right)}$

**Table 2:** Equations for secant stiffness matrices.

Shearing Strain	Crack Surface Slipping Down a Slope	Crack Surface Is Not Slipping	Crack Slipping Up a Slope
positive total shearing strain (*γ*_12_ > 0)	$\tau_{12}^{a}=\frac{\beta^{\prime }G\gamma_{12}+\mu^{down}\left(E_{c}\varepsilon_{eff}\right)}{\left(1+\beta^{\prime }\right)}=\frac{\beta^{\prime }G\gamma_{12}+\mu^{down}E_{c}\left(\varepsilon_{1}-\frac{\gamma_{12}}{a}\right)}{\left(1+\beta^{\prime }\right)}$	$\tau_{12}^{b}=\tau_{12,prev}+G\left(\gamma_{12}-\gamma_{12,prev}\right)$	$\tau_{12}^{c}=\frac{\beta^{\prime }G\gamma_{12}-\mu^{up}\left(E_{c}\varepsilon_{eff}\right)}{\left(1+\beta^{\prime }\right)}=\frac{\beta^{\prime }G\gamma_{12}-\mu^{up}E_{c}\left(\varepsilon_{1}-\frac{\gamma_{12}}{a}\right)}{\left(1+\beta^{\prime }\right)}$
negative total shearing strain (*γ*_12_ < 0)	$\tau_{12}^{c}=\frac{\beta^{\prime }G\gamma_{12}-\mu^{down}\left(E_{c}\varepsilon_{eff}\right)}{\left(1+\beta^{\prime }\right)}=\frac{\beta^{\prime }G\gamma_{12}-\mu^{down}E_{c}\left(\varepsilon_{1}+\frac{\gamma_{12}}{a}\right)}{\left(1+\beta^{\prime }\right)}$	$\tau_{12}^{b}=\tau_{12,prev}+G\left(\gamma_{12}-\gamma_{12,prev}\right)$	$\tau_{12}^{a}=\frac{\beta^{\prime }G\gamma_{12}+\mu^{up}\left(E_{c}\varepsilon_{eff}\right)}{\left(1+\beta^{\prime }\right)}=\frac{\beta^{\prime }G\gamma_{12}+\mu^{up}E_{c}\left(\varepsilon_{1}+\frac{\gamma_{12}}{a}\right)}{\left(1+\beta^{\prime }\right)}$

**Table 3:** Secant stiffness matrices at crack direction 1 (active).

Shearing Strain	Crack Surface Slipping Down a Slope	Crack Surface Is Not Slipping	Crack Slipping Up a Slope
positive total shearing strain (*γ*_12_ > 0)	$\left\{\begin{array}{c} \sigma_{1}\\ \sigma_{2}\\ \tau_{12} \end{array}\right\}=\left[\begin{array}{ccc} E_{c} & 0 & -\frac{E_{c}}{a}\\ 0 & E_{c} & 0\\ \frac{\mu^{down}E_{c}}{1+\beta^{\prime }} & 0 & \frac{\beta^{\prime }G-\mu^{down}E_{c}/a}{1+\beta^{\prime }} \end{array}\right]\left\{\begin{array}{c} \varepsilon_{1}\\ \varepsilon_{2}\\ \gamma_{12} \end{array}\right\}$	$\left\{\begin{array}{c} \sigma_{1}\\ \sigma_{2}\\ \tau_{12} \end{array}\right\}=\left[\begin{array}{ccc} E_{c} & 0 & -\frac{E_{c}}{a}\\ 0 & E_{c} & 0\\ 0 & 0 & \frac{\tau_{12}^{b}}{\gamma_{12}^{\mathrm{old}}} \end{array}\right]\left\{\begin{array}{c} \varepsilon_{1}\\ \varepsilon_{2}\\ \gamma_{12} \end{array}\right\}$	$\left\{\begin{array}{c} \sigma_{1}\\ \sigma_{2}\\ \tau_{12} \end{array}\right\}=\left[\begin{array}{ccc} E_{c} & 0 & -\frac{E_{c}}{a}\\ 0 & E_{c} & 0\\ \frac{-\mu^{up}E_{c}}{1+\beta^{\prime }} & 0 & \frac{\beta^{\prime }G+\mu^{up}E_{c}/a}{1+\beta^{\prime }} \end{array}\right]\left\{\begin{array}{c} \varepsilon_{1}\\ \varepsilon_{2}\\ \gamma_{12} \end{array}\right\}$
negative total shearing strain (*γ*_12_ < 0)	$\left\{\begin{array}{c} \sigma_{1}\\ \sigma_{2}\\ \tau_{12} \end{array}\right\}=\left[\begin{array}{ccc} E_{c} & 0 & \frac{E_{c}}{a}\\ 0 & E_{c} & 0\\ \frac{-\mu^{down}E_{c}}{1+\beta^{\prime }} & 0 & \frac{\beta^{\prime }G-\mu^{down}E_{c}/a}{1+\beta^{\prime }} \end{array}\right]\left\{\begin{array}{c} \varepsilon_{1}\\ \varepsilon_{2}\\ \gamma_{12} \end{array}\right\}$	$\left\{\begin{array}{c} \sigma_{1}\\ \sigma_{2}\\ \tau_{12} \end{array}\right\}=\left[\begin{array}{ccc} E_{c} & 0 & \frac{E_{c}}{a}\\ 0 & E_{c} & 0\\ 0 & 0 & \frac{\tau_{12}^{b}}{\gamma_{12}^{\mathrm{old}}} \end{array}\right]\left\{\begin{array}{c} \varepsilon_{1}\\ \varepsilon_{2}\\ \gamma_{12} \end{array}\right\}$	$\left\{\begin{array}{c} \sigma_{1}\\ \sigma_{2}\\ \tau_{12} \end{array}\right\}=\left[\begin{array}{ccc} E_{c} & 0 & \frac{E_{c}}{a}\\ 0 & E_{c} & 0\\ \frac{\mu^{up}E_{c}}{1+\beta^{\prime }} & 0 & \frac{\beta^{\prime }G+\mu^{up}E_{c}/a}{1+\beta^{\prime }} \end{array}\right]\left\{\begin{array}{c} \varepsilon_{1}\\ \varepsilon_{2}\\ \gamma_{12} \end{array}\right\}$

**Table 4:** Secant stiffness matrices at crack direction 2 (active).

Shearing Strain	Crack Surface Slipping Down a Slope	Crack Surface Is Not Slipping	Crack Slipping Up a Slope
positive total shearing strain (*γ*_12_ > 0)	$\left\{\begin{array}{c} \sigma_{1}\\ \sigma_{2}\\ \tau_{12} \end{array}\right\}=\left[\begin{array}{ccc} E_{c} & 0 & 0\\ 0 & E_{c} & -\frac{E_{c}}{a}\\ 0 & \frac{\mu^{down}E_{c}}{1+\beta^{\prime }} & \frac{\beta^{\prime }G-\mu^{down}E_{c}/a}{1+\beta^{\prime }} \end{array}\right]\left\{\begin{array}{c} \varepsilon_{1}\\ \varepsilon_{2}\\ \gamma_{12} \end{array}\right\}$	$\left\{\begin{array}{c} \sigma_{1}\\ \sigma_{2}\\ \tau_{12} \end{array}\right\}=\left[\begin{array}{ccc} E_{c} & 0 & 0\\ 0 & E_{c} & -\frac{E_{c}}{a}\\ 0 & 0 & \frac{\tau_{12}^{b}}{\gamma_{12}^{\mathrm{old}}} \end{array}\right]\left\{\begin{array}{c} \varepsilon_{1}\\ \varepsilon_{2}\\ \gamma_{12} \end{array}\right\}$	$\left\{\begin{array}{c} \sigma_{1}\\ \sigma_{2}\\ \tau_{12} \end{array}\right\}=\left[\begin{array}{ccc} E_{c} & 0 & 0\\ 0 & E_{c} & -\frac{E_{c}}{a}\\ 0 & \frac{-\mu^{up}E_{c}}{1+\beta^{\prime }} & \frac{\beta^{\prime }G+\mu^{up}E_{c}/a}{1+\beta^{\prime }} \end{array}\right]\left\{\begin{array}{c} \varepsilon_{1}\\ \varepsilon_{2}\\ \gamma_{12} \end{array}\right\}$
negative total shearing strain (*γ*_12_ < 0)	$\left\{\begin{array}{c} \sigma_{1}\\ \sigma_{2}\\ \tau_{12} \end{array}\right\}=\left[\begin{array}{ccc} E_{c} & 0 & 0\\ 0 & E_{c} & \frac{E_{c}}{a}\\ 0 & \frac{-\mu^{down}E_{c}}{1+\beta^{\prime }} & \frac{\beta^{\prime }G-\mu^{down}E_{c}/a}{1+\beta^{\prime }} \end{array}\right]\left\{\begin{array}{c} \varepsilon_{1}\\ \varepsilon_{2}\\ \gamma_{12} \end{array}\right\}$	$\left\{\begin{array}{c} \sigma_{1}\\ \sigma_{2}\\ \tau_{12} \end{array}\right\}=\left[\begin{array}{ccc} E_{c} & 0 & 0\\ 0 & E_{c} & \frac{E_{c}}{a}\\ 0 & 0 & \frac{\tau_{12}^{b}}{\gamma_{12}^{\mathrm{old}}} \end{array}\right]\left\{\begin{array}{c} \varepsilon_{1}\\ \varepsilon_{2}\\ \gamma_{12} \end{array}\right\}$	$\left\{\begin{array}{c} \sigma_{1}\\ \sigma_{2}\\ \tau_{12} \end{array}\right\}=\left[\begin{array}{ccc} E_{c} & 0 & 0\\ 0 & E_{c} & \frac{E_{c}}{a}\\ 0 & \frac{\mu^{up}E_{c}}{1+\beta^{\prime }} & \frac{\beta^{\prime }G+\mu^{up}E_{c}/a}{1+\beta^{\prime }} \end{array}\right]\left\{\begin{array}{c} \varepsilon_{1}\\ \varepsilon_{2}\\ \gamma_{12} \end{array}\right\}$

**Table 5:** Summary of active crack criteria.

^e^ε_1_	^e^ε_2_	Crack 1 Surface State	Crack 2 Surface State	Active Crack
+	+	Tension	Tension	Split—both cracks
+	−	Tension	Compression	Crack 1
−	+	Compression	Tension	Crack 2
−	−	Compression	Compression	Requires analysis of shear-stress limits

**Table 6:** Material properties and parameters for the base model.

Material Properties and Parameters	Values
Concrete Compressive Strength	41.4 MPa
Concrete Modulus of Elasticity (*E_c_*)	27.6 Gpa
Concrete Shear Modulus (*G*)	13.8 Gpa
Shear Retention Factor (*β′*)	0.05
Steel Yield Strength	414 Mpa
Steel Modulus of Elasticity (*E_s_*)	206.8 Gpa
Friction Coefficients (*µ_up_*)	1.2
Friction Coefficients (*µ_down_*)	0.2
Crack Opening Path (*a_cop_*)	2.0

## Data Availability

Data is contained within the article.

## Notations

The following notations are used in this manuscript:

*a*	shape vector multiplier
*a_cop_*	a parameter that defines the slope of the crack opening path
*f*_cr_	concrete crack stress
*E_c_*	modulus of elasticity of concrete
*G*	concrete shear modulus
*N*	concrete normal strength
*V*	concrete shear strength
*β*′	dowel action shear retention factor
*ε_cr_*	concrete crack strain
*^e^ε*	effective concrete crack strain
*^e^ε*_1_	effective concrete crack strain in the 1 direction
*^e^ε*_2_	effective concrete crack strain in the 2 direction
*^e^ε^A^_cr_*	effective concrete crack strain at Point A
*ε*_1_	concrete crack strain in the 1 direction
*ε*_2_	concrete crack strain in the 2 direction
*γ*_12_	shear strain
*γ*_12,_*_ prev_*	previously converged shear strain
*µ′*	friction coefficient defined relative to the crack opening path
*µ^up^*	friction coefficient defined relative to crack surface in the upslope direction
*µ^down^*	friction coefficient defined relative to crack surface in the downslope direction
*σ*_1_	concrete stress in the 1 direction
*σ*_2_	concrete stress in the 2 direction
*σ_xy, prev_*	previously converged concrete stress in the x direction
*τ*_12_	shear stress
*τ^a^*_12_	minimum shear stress
*τ^b^*_12_	outside upper limit shear stress
*τ^c^*_12_	maximum shear stress
*τ*_12, _*_prev_*	previously converged shear stress
A	global actions or forces
S	global stiffness matrix
D	nodal displacements
Ã,$\;\tilde{B}\;$	shape vectors
